# Combined Transcriptome and Metabolome Analyses of Oxidative Stress Regulatory Mechanism in Porcine Follicular Granulosa Cells

**DOI:** 10.3390/biology14111519

**Published:** 2025-10-30

**Authors:** Xilin Bi, Shu Niu, Yu Zhang, Qiang Liu, Qihang Zhang, Ruirong Hao

**Affiliations:** 1 College of Animal Science, Shanxi Agricultural University, Taigu 030801, China; bixilin9645@163.com (X.B.); niushu1387@163.com (S.N.); 16634201564@163.com (Y.Z.); 15235962856@163.com (Q.L.); zqh15253126100@outlook.com (Q.Z.); 2 Key Laboratory of Farm Animal Genetic Resources Exploration and Breeding of Shanxi Province, Taigu 030801, China

**Keywords:** transcriptomics, untargeted metabolomics, conjoint analysis, oxidative stress

## Abstract

Follicular atresia is mainly driven by the oxidative stress-induced apoptosis of granulosa cells (GCs). Oxidative stress mediated by H_2_O_2_ is the predominant form of stress in cells and plays a key role in the death of porcine GCs. In the present study, using integrated transcriptomic and untargeted metabolomic approaches, we explored the mechanisms underlying the regulation of oxidative stress in porcine follicular GCs. Per the transcriptomic analysis, compared with the control group, we identified 328 differentially expressed mRNAs (260 upregulated, 68 downregulated) in the H_2_O_2_-treatment group; these mRNAs were significantly enriched in apoptosis-related pathways, including the tumour necrosis factor (TNF) and p53 signalling pathways. Furthermore, via untargeted metabolomic analysis, we identified 150 differentially expressed metabolites (101 positive, 49 negative). The pathways associated with protein digestion and absorption, glycine, serine, and threonine metabolism, amino acid biosynthesis, and carbon metabolism were enriched with these metabolites. The integrated transcriptomic and metabolomic analyses revealed taurine, creatine, L-serine, and hypoxanthine as the key metabolites under H_2_O_2_-induced oxidative stress. Both the differential genes and metabolites were notably enriched in the FOXO and mineral absorption pathways. In the present study, we elucidated the regulatory mechanism underlying H_2_O_2_-induced oxidative stress in porcine follicular GCs via transcriptomic and metabolomic analyses. Our findings offer novel insights into the alleviation of oxidative stress in GCs.

## Simple Summary

This study investigates how oxidative stress leads to the death of granulosa cells, which are essential for egg development in pigs. Such cell death is a key factor causing follicular degeneration and reduced fertility. The researchers exposed these cells to hydrogen peroxide, a common source of cellular stress, and analyzed the resulting changes in gene activity and small molecule production. They found 328 genes whose activity changed significantly, many linked to processes that control cell death. In addition, 150 chemical compounds within the cells were altered, mainly those involved in energy production and amino acid metabolism. Further analysis identified several important molecules—taurine, creatine, serine, and hypoxanthine—that appear to help regulate the cell’s response to oxidative stress. The findings suggest that these molecules and related biological pathways may play protective roles in maintaining cell health. Overall, this study provides new insights into how oxidative stress affects reproductive cells and could guide strategies to improve animal fertility and potentially human reproductive health by reducing stress-related cell damage.

## 1. Introduction

Oxidative stress arises when there is an imbalance between reactive oxygen species (ROS) and the cellular antioxidant defense system, leading to ROS generation at levels exceeding the normal concentration required for cellular homeostasis [1]. ROS encompass oxygen free radicals, including superoxide anion ($O_{2}^{-}$) and hydroxyl (·OH) radicals, as well as non-radical oxidants, such as hydrogen peroxide (H_2_O_2_) and singlet oxygen (^1^O_2_) [2]. ROS react chemically with DNA, proteins, lipids, and other cellular molecules [3]. However, increased ROS levels can lead to DNA damage, protein modifications, and lipid peroxidation, ultimately resulting in oxidative stress and cell death [4]. In the follicular microenvironment, excessive ROS disrupts the antioxidant balance, leading to decreased levels of glutathione, superoxide dismutase (SOD), and catalase (CAT), thereby compromising the stability of the follicular fluid. Concurrently, ROS reduces mitochondrial membrane potential and ATP production in oocytes, impairing their maturation and fertilization competence, while activating the mitochondrial apoptotic pathway, which damages theca and granulosa cells and contributes to premature follicular atresia [5]. Moreover, ROS inhibits the expression of aromatase and key steroidogenic enzymes such as CYP11A1 and CYP17A1, resulting in insufficient synthesis of estrogen, androgen, and progesterone, ultimately hindering follicular development. In addition, oxidative stress disrupts communication between theca cells, granulosa cells, and oocytes, thereby impairing the functional integrity of the follicle–oocyte complex [6].

Among the known ROS, H_2_O_2_ is produced intracellularly and is considered to be a less reactive and more stable species. Therefore, H_2_O_2_ can serve as an important intracellular and intercellular signalling molecule [7]. H_2_O_2_, as a signalling molecule, can diffuse through cells and tissues, triggering swift cellular responses, including alterations in cell morphology, the onset of cell proliferation, and the recruitment of immune cells [8]. Exposure to H_2_O_2_ induces oxidative stress in cellular models. The reaction between Fenton reagents (Fe^2+^ and H_2_O_2_) generates highly reactive hydroxyl radicals; this is considered the main mechanism underlying oxidative damage [9]. Studies have revealed that the concentrations of H_2_O_2_ required to induce oxidative damage in cellular models are higher than those observed in vivo. The concentrations typically used extracellularly (often in the range of several hundred micromolar) are substantially higher than the corresponding intracellular levels, which usually remain in the low micromolar range. This is because H_2_O_2_ enters the cell via passive diffusion, a process limited by its membrane permeability, resulting in a lower intracellular concentration compared to the extracellular environment. Moreover, the intracellular milieu possesses a robust antioxidant enzyme system, including catalase and glutathione peroxidase, which rapidly decompose H_2_O_2_ into water and oxygen, further reducing its intracellular levels. When high concentrations of H_2_O_2_ are artificially applied extracellularly, a substantial portion is either degraded before crossing the cell membrane or immediately upon entry, leading to an effective intracellular concentration typically in the micromolar range [10]. H_2_O_2_ acts directly on cells to produce oxygen (O_2_) via catalase activity [11,12]. Therefore, inducing oxidative stress using H_2_O_2_ typically requires high micromolar concentrations of H_2_O_2_ [10].

Transcriptome sequencing analyses the collective RNA produced by specific tissues or cells in a particular state, enabling the exploration of gene function at the mRNA level. This approach revealed the molecular mechanisms underlying specific biological processes and disease development. In contrast, untargeted metabolomic sequencing investigates dynamic shifts in small-molecule metabolites in response to stimuli or perturbations in cells, tissues, organs, or organisms, both before and after intervention. The physiological mechanisms underlying these changes can be clarified by identifying differential metabolites and performing pathway enrichment analysis. Oxidative stress is a complex biological process, and single-omics analyses often fail to comprehensively elucidate the regulatory mechanisms of such intricate physiological processes. Multiomics analysis, which integrates transcriptomics and metabolomics, addresses these limitations by providing a deeper and multifaceted perspective. This integration enables the investigation of biological questions from both causative and consequential perspectives, offering mutual validation and broader insights.

This study advances understanding of oxidative stress mechanisms in ovarian physiology by integrating transcriptomic and metabolomic analyses to achieve a system-level view. Unlike previous single-omics studies that focused on ROS-induced apoptosis or steroidogenic disruption, this research elucidates the transcription–metabolism interplay underlying H_2_O_2_-induced oxidative stress in porcine granulosa cells. Through multi-omics integration, it identifies key transcriptional regulators, metabolites, and pathways that mediate cellular redox imbalance, amino acid metabolism, and steroid biosynthesis. We hypothesize that H_2_O_2_ triggers coordinated transcriptional and metabolic reprogramming driven by specific transcription factors and metabolic enzymes involved in redox regulation and energy homeostasis. This integrative framework not only reveals molecular signatures of oxidative stress but also provides new insights into the systemic regulation of follicular function. The findings establish a foundation for developing targeted strategies to preserve granulosa cell viability and improve oocyte quality under oxidative conditions.

## 2. Materials and Methods

### 2.1. Porcine GCs Culture In Vitro

The ovaries used in this study were obtained from the Kaiyuan Meat Industry Slaughterhouse in Taigu County, Shanxi Province, China. The experimental design and procedures were carried out in accordance with the guidelines of Experimental Animal Ethical Committees of Shanxi Agricultural University (permit no. SXAU-EAW-2023P.OP.009010207). During the spring and autumn seasons, freshly excised ovaries were placed in pre-chilled sterile phosphate-buffered saline (PBS) (Servicebio, Wuhan, China) and transported to the laboratory for processing within one hour. GCs isolation was subsequently performed under a laminar flow hood in a sterile cell culture room. The follicular fluid and follicular wall GCs were collected from porcine follicles with diameters ranging from 3 to 6 mm, centrifuged, washed, and used for GCs isolation. The GCs were cultured in DMEM/F12 medium (Thermo Fisher Scientific, Waltham, MA, USA) supplemented with 10% foetal bovine serum (Dcell, Shanghai, China) and 1% penicillin-streptomycin (Thermo Fisher Scientific, Waltham, MA, USA).

### 2.2. H_2_O_2_ Treatment

H_2_O_2_ (Sigma-Aldrich, St. Louis, MO, USA) was used to induce oxidative stress in the cells. Porcine GCs were treated with various concentrations of H_2_O_2_ (0, 50, 100, 150, 200, 300, 400, and 500 μM) for different durations (2, 4, and 6 h). Various studies, such as cell viability assays, ROS detection, and morphological evaluation, were conducted to confirm that H_2_O_2_ induces ROS accumulation without deleterious effects on GCs.

### 2.3. Cell Viability Analysis

Cell proliferation assays were performed using CCK-8 (Dojindo, Shanghai, China). When GCs seeded in 96-well plates reached approximately 90% confluency, they were treated with H_2_O_2_ or DMEM/F12 medium, then 10 μL CCK-8 was added to each well and incubated in 5% CO_2_ at 37 °C for 4 h. The absorbance (optical density, OD) was measured at 450 nm using a microplate reader. Wells containing culture medium without cells were used as a blank for normalization. The biological replicate number is three.

### 2.4. ROS Detection

ROS levels in porcine GCs post-H_2_O_2_ treatment were determined using the ROS assay kit (Beyotime, Shanghai, China). Porcine GCs were treated with H_2_O_2_ and washed three times with PBS. The 2′, 7′-dichlorodihydrofluorescein diacetate (DCFH-DA) was diluted with serum-free DMEM/F12 medium at 1:1000 to ensure a final concentration of 10 μM. The GCs were incubated in the dark with DCFH-DA (10 μM) at 37 °C for 20 min. After incubation, they were washed three times with an FBS-free medium. The ROS levels in porcine GCs treated with H_2_O_2_ were detected using fluorescence microscopy. The biological replicate number is three.

### 2.5. Sample Collection for Sequencing

The cells were cleaned with PBS and collected using a cell scraper. Cells immersed in PBS were centrifuged at 1000× *g* for 5 min at 4 °C to obtain the cells, finally stored at −80 °C until further use for transcriptomic and metabolomics analyses. Each group consisted of three biological replicates for transcriptomic samples and six biological replicates for metabolomics samples.

### 2.6. RNA Extraction, Library Construction, and Sequencing

Total RNA from the three H_2_O_2_ treatment and three control groups was extracted using TRIzol (Takara, Dalian, China) reagent following the manufacturer’s instructions. RNA was then quantified using an Agilent 2100 Bioanalyzer (Thermo Fisher Scientific, Waltham, MA, USA). First, rRNA was removed from the total RNA at a certain temperature in an ionic environment. The first strand of cDNA was synthesised using random primers and reverse transcriptase. The second strand was synthesised using DNA polymerase I and RNaseH. During the second-strand synthesis of the cDNA, the RNA templates were removed. The PCR product was amplified and thermally denatured into a single strand, which was cyclized with a splint oligo to obtain a single-stranded circular DNA library. Finally, the constructed cDNA libraries were subjected to paired-end sequencing with a paired-end 100 bp (PE100) reading length on a BGIseq500 platform (BGI, Shenzhen, China).

### 2.7. Transcriptomic Data Analysis

Clean data (clean reads) were obtained by removing low-quality reads from the raw data. Then the clean reads were aligned with the reference genome (GCF_000003025.6_Sscrofa11.1) using HISAT2 v2.1.0 [13]. Gene expression level across samples was calculated based on the FPKM value for each sample. The mRNAs were counted only if at least one group of three samples with an average gene expression greater than three was present. The intracellular gene expression differences between granulosa cells treated with H_2_O_2_ and those in the control group were evaluated based on fold-change values calculated from the raw count data using DESeq2 v3.15 [14]. DE mRNAs were defined as genes exhibiting a fold-change (FC) > 2 and *Q* value < 0.05. Subsequently, functional annotation of DE mRNAs was conducted using Gene Ontology (GO) and Kyoto Encyclopedia of Genes and Genomes (KEGG) enrichment analyses, implemented via the phyper function in R (v3.6.3).

### 2.8. Metabolite Extraction and LC-MS/MS Analysis

The cell samples were gradually thawed at 4 °C and processed according to the manufacturer’s instructions. For quality control (QC) purposes, 20 μL of the supernatant from each sample was pooled to generate a QC sample, which was used to evaluate the reproducibility and stability of the LC-MS analytical procedure. Liquid chromatography–tandem mass spectrometry (LC-MS/MS) analysis was performed using a BEH C18 chromatographic column (1.7 μm, 2.1 × 100 mm) (Waters, Milford, MA, USA). High-resolution mass spectrometry data acquisition (MS1 and MS2) was conducted on a Q Exactive mass spectrometer (Thermo Fisher Scientific, Waltham, MA, USA). The raw LC-MS/MS data files (in raw format) were imported into Compound Discoverer 3.1 (Thermo Fisher Scientific, Waltham, MA, USA) for data processing. Key processing steps included peak extraction, retention time alignment within and between groups, adduct ion merging, missing value imputation, background peak annotation, and metabolite identification. The resulting data included molecular weight, retention time, peak area, and identification results for the detected compounds.

### 2.9. Metabolomic Data Analysis

The MS metabolite identification was performed by cross-referencing the Borland Graphics Interface (BGI) Library, mzCloud, and ChemSpider databases. Each sample was clustered based on Principal Coordinates Analysis (PCA). Partial least squares-discriminant analysis (PLS-DA) [15] were performed to develop a relationship model between metabolite expression levels and samples. Differential metabolites between the H_2_O_2_ treatment group and the control group were then identified based on fold change (FC) and t-test analysis. The selection criteria for differential metabolites were: (1) a VIP score of the first two principal components from the PLS-DA model ≥ 1, FC ≥ 1.2 or ≤0.83, and a *p* value < 0.05. Functional annotation of the DE metabolites was conducted using the KEGG database.

### 2.10. Combined Transcriptome and Metabolome Analyses

The regularized canonical correlation analysis (rCCA) was performed to evaluate the relationships between differentially expressed genes and metabolites. Prior to analysis, both datasets were log-transformed and standardized. The regularization parameters (λ_1_ and λ_2_) were optimized through a grid search combined with 5-fold cross-validation, selecting the combination yielding the highest mean canonical correlation coefficient. Model robustness was further assessed by bootstrap resampling to ensure stability of canonical loadings. The analysis was implemented using the *mixOmics* R package (v6.24.0). [16]. whereafter, the block.splsda function [17] was used to analyse the differential genes and metabolites. MetaboAnalyst 6.0 (https://www.metaboanalyst.ca/) (accessed on 5 May 2024) was used to perform interaction network analysis on the differential genes and metabolites. Construct a simplified regulatory pathway map using the pathway network data provided by the Pathview package in R (v3.6.3).

### 2.11. qRT-PCR Verification of DEmRNAs

Eight genes (*PLK2*, *LIPG*, *ATF4*, *TP53INP1*, *HMOX1*, *BTG1*, *SLC40A1*, and *TXNIP*) were randomly selected from the DE mRNAs for validation of the RNA-Seq results using quantitative real-time PCR (qRT-PCR). Total RNA was extracted using RNAiso Plus (Takara, Dalian, China), and complementary DNA (cDNA) was synthesized with the PrimeScript™ RT Reagent Kit with gDNA Eraser (Takara, Dalian, China). The primers were synthesized by Sangon Biotech (Shanghai, China), and their detailed sequences are provided in Supplementary File S1. qRT-PCR reactions were performed using TB Green^®^ Premix Ex Taq™ II (Takara, Dalian, China) on a CFX Connect™ Real-Time PCR Detection System (Bio-Rad, Hercules, CA, USA). Three biological replicates were conducted, with each biological replicate containing three technical replicates. GAPDH served as the internal reference gene, and the relative expression levels of target genes were calculated using the 2^−ΔΔCt^ method.

### 2.12. Statistical Analysis

Statistical analyses were performed using GraphPad Prism 8.0 (GraphPad Software, San Diego, CA, USA) and SPSS 22.0 (SPSS Software, Chicago, IL, USA). Significance of differences was determined using the *t*-test, with *p* < 0.05 considered statistically significant, while “ns” indicated no significant difference. The significance levels were denoted as *p* < 0.05, *p* < 0.01, and *p* < 0.001.

## 3. Results

### 3.1. Construction of an Oxidative Stress Model of Porcine GCs Cultured In Vitro

**Figure 1:**
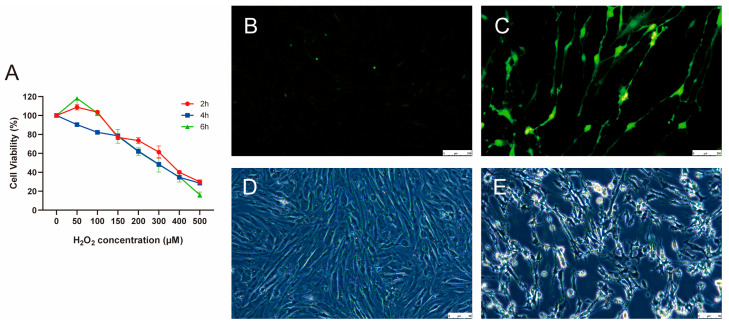
Construction of oxidative stress model of porcine GCs. (**A**) Change in cell viability after 2, 4, and 6 h of treatment with various concentrations of H_2_O_2_. The biological replicate number is three. (**B**,**C**) ROS levels in control and 300 µM H_2_O_2_-treatment groups for 4 h, respectively. The biological replicate number is three. Scale: 250 µM. (**D**,**E**) The morphological characteristics of porcine GCs in control and 300 µM H_2_O_2_-treatment groups for 4 h, respectively. Data is represented as mean ± SEM. GCs, granulosa cells, ROS, reactive oxygen species. Scale: 50 µM.

The primary cultured porcine GCs were treated with various concentrations of H_2_O_2_ (0, 50, 100, 150, 200, 300, 400, and 500 µM) for different durations: 2, 4, and 6 h. H_2_O_2_ significantly reduced cell viability in a dose- and time-dependent manner (Figure 1A). Compared with the H_2_O_2_-treatment groups at 2 and 6 h, the viability of cells in the treatment groups at 4 h decreased with increasing H_2_O_2_ concentrations. After treatment with 300 µM H_2_O_2_ for 4 h, the cell viability was approximately 50%, which was considered the threshold for cellular oxidative stress. Subsequently, ROS levels in control and 300 µM H_2_O_2_-treatment groups for 4 h were assessed (Figure 1B,C). Compared to the control group, after 4 h of treatment with 300 μM H_2_O_2_, the ROS levels were significantly upregulated, indicating excessive ROS production and accumulation. Microscopic observation of the porcine GCs revealed that after treatment with 300 μM H_2_O_2_ for 4 h, the cells exhibited morphological changes, including cell atrophy and jagged edges, indicating that the porcine GCs had lost their membrane integrity and vitality (Figure 1D,E). These observations suggest that we successfully established an oxidative stress model of porcine GCs cultured in vitro by treating GCs with 300 μM H_2_O_2_ for 4 h.

### 3.2. Quality Control of RNA-Sequencing

The quality of RNA extracted from porcine GCs in both the control and H_2_O_2_ treatment groups was assessed using the Agilent 2100 Bioanalyzer (Agilent Technologies, Santa Clara, CA, USA). At a 5-fold dilution, the total RNA concentrations ranged from 100 to 200 ng/µL. The RNA integrity numbers (RIN) for all samples were ≥8.0, and the ribosomal RNA (28S:18S) ratios were ≥1.5 (see Supplementary File S2). These results indicate that the RNA samples exhibited high purity and integrity, thereby meeting the quality requirements for subsequent RNA sequencing (RNA-seq) analysis. To identify potential transcripts in porcine GCs, three GC samples were collected from different groups (H_2_O_2_ treatment and control). We obtained 167–184 million and 147–173 million unique mapped clean reads from the H_2_O_2_-treated and control libraries, respectively (see Supplementary File S3). After quality control, the Q20 and Q30 read percentages were 97.12% and 92.72%, respectively (Supplementary File S2). Compared with the reference genome Sscrofa11.1, the mapping ratio for each sample exceeded 85.68% (Supplementary File S3). In conclusion, these findings validate that both the samples and the sequencing data utilized in this study were of high quality and reliability.

### 3.3. Analysis of Differences Between Control and H_2_O_2_ Groups

**Figure 2:**
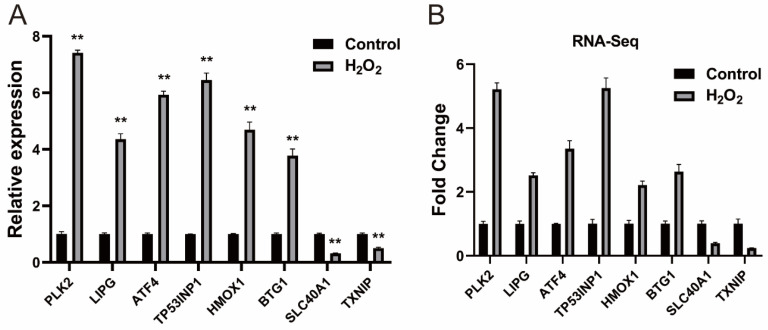
Verification of the DE mRNAs (**A**) Verification of DE mRNAs by qRT-PCR; (**B**) Eight mRNAs were selected from the RNA-Seq data. The qRT-PCR data are presented as the average of three values ± SEM. ** *p* < 0.01.

We performed a difference analysis according to the methods section. Compared with the control group, we identified 328 (260 upregulated and 68 downregulated) differentially expressed (DE) mRNAs, the detailed information for which is provided in Supplementary Files S4 and S5. DE mRNAs were subjected to cluster analysis, and the results are presented in Supplementary File S6. Eight DE mRNAs were selected for qRT-PCR validation, and the results were consistent with those obtained from RNA-seq, confirming the reliability of the RNA-seq data (Figure 2).

### 3.4. GO and KEGG Analysis

**Figure 3:**
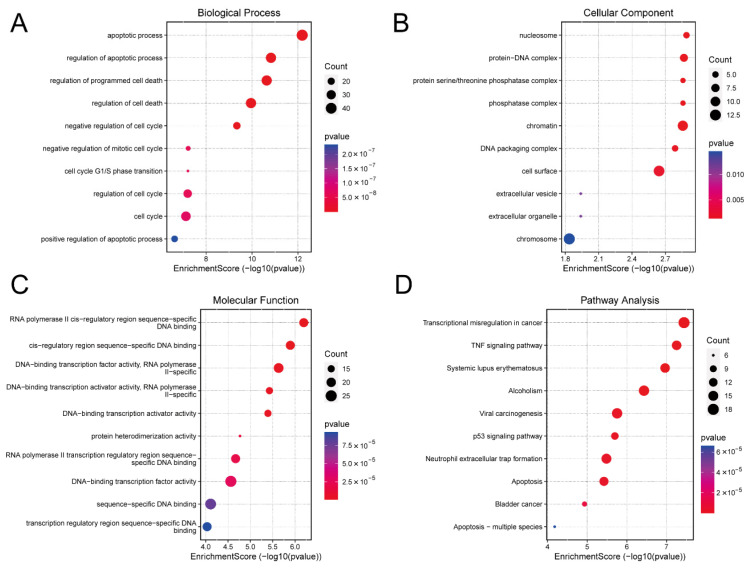
Functional Enrichment Analysis. (**A**) Biological process of DE mRNAs. (**B**) Cellular component of DE mRNAs. (**C**) Molecular function of DE mRNAs. (**D**) The KEGG pathway bubble chart of DE mRNAs. DE, differentially expressed; KEGG, Kyoto Encyclopaedia of Genes and Genomes.

Gene Ontology (GO) analysis indicated that the DE mRNAs were predominantly involved in biological processes associated with apoptosis and cell cycle. DNA-binding transcription factor activity was particularly enriched in the molecular function category (Figure 3A–C). Kyoto Encyclopedia of Genes and Genomes (KEGG) pathway analysis further highlighted the enrichment of DE mRNAs in the tumour necrosis factor (TNF), p53, and apoptosis signaling pathways (Figure 3D). These pathways play a key role in cell survival, proliferation, differentiation, necrosis, and apoptosis. The detailed information for which is provided in Supplementary File S7.

### 3.5. Data Quality of Untargeted Metabolomics Sequencing

Data quality was evaluated by examining the repeatability of the quality control (QC) sample detection. This assessment included the overlap of the base peak chromatogram (BPC) for the QC samples (Supplementary File S8A,B), PCA (Supplementary File S8C,D), and coefficient of variation (CV) profiles for the compounds (Supplementary File S8E,F) in each group. The results demonstrated stable signal consistency throughout the sample detection and analysis, with the QC samples showing strong clustering, thereby confirming that the data quality was reliable and acceptable.

### 3.6. Identification and Classification of Metabolites

**Figure 4:**
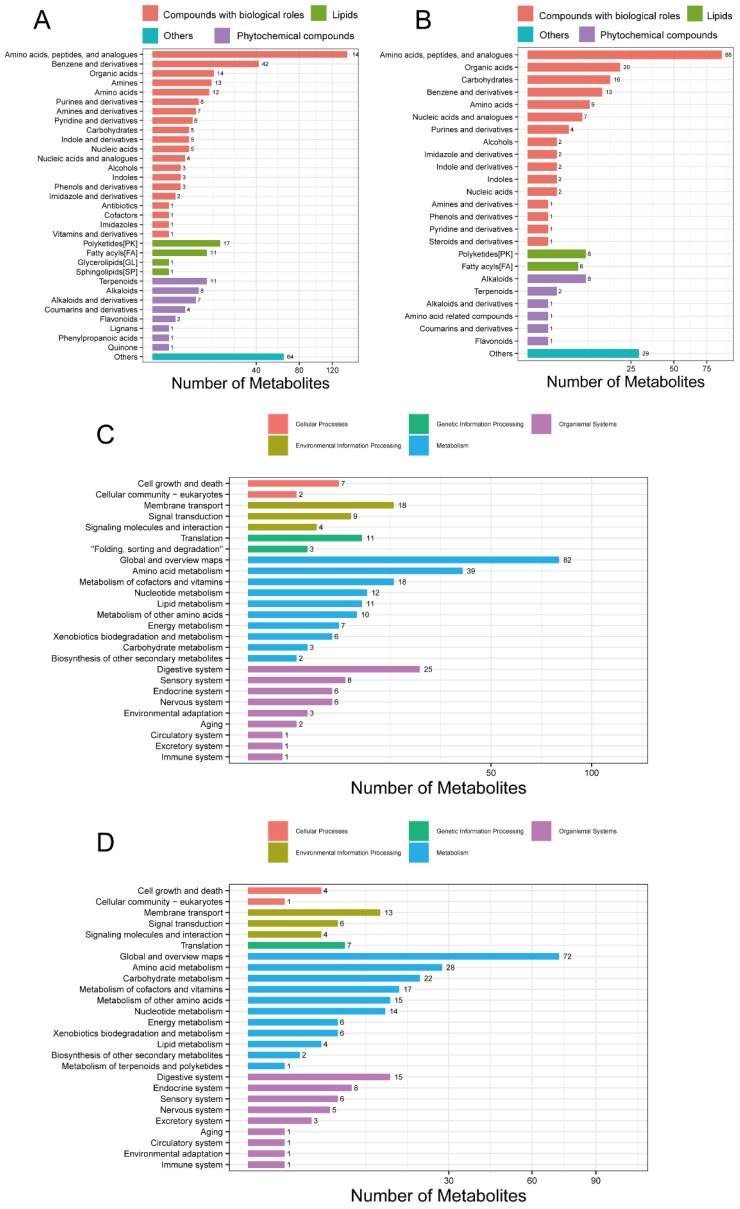
Classification and KEGG analysis of metabolites. (**A**) Metabolite classification from the POS mode. (**B**) Metabolite classification from the NEG mode. (**C**) KEGG function from the POS mode. (**D**) KEGG function from the NEG mode. POS, positive ion mode; NEG, negative ion mode; KEGG, Kyoto Encyclopaedia of Genes and Genomes.

After data quality control, a total of 2316 compounds were identified in the positive ion mode (POS) and 946 in the negative ion mode (NEG). The breakdown of the identified compounds was: 698 in the POS and 364 in the NEG. The identified POS- (Figure 4A) and NEG-specific (Figure 4B) metabolites were classified and annotated using the KEGG database and the Human Metabolome Database (HMDB). It was found that amino acids, peptides, and their analogues, polyketides, and alkaloids were more abundant than other metabolites. Functional annotations of the identified POS (Figure 4C) and NEG (Figure 4D) metabolites were performed using the KEGG database, which revealed that both the POS- and NEG-specific metabolites were enriched in pathways related to amino acid, cofactor, vitamin, and nucleotide metabolism. In addition, the POS-specific metabolites were enriched in lipid and other amino acid metabolism. The NEG-specific metabolites were enriched in carbohydrate and other amino acid metabolism. The detailed information for which is provided in Supplementary Files S9 and S10.

### 3.7. Screening of Differential Metabolites

**Figure 5:**
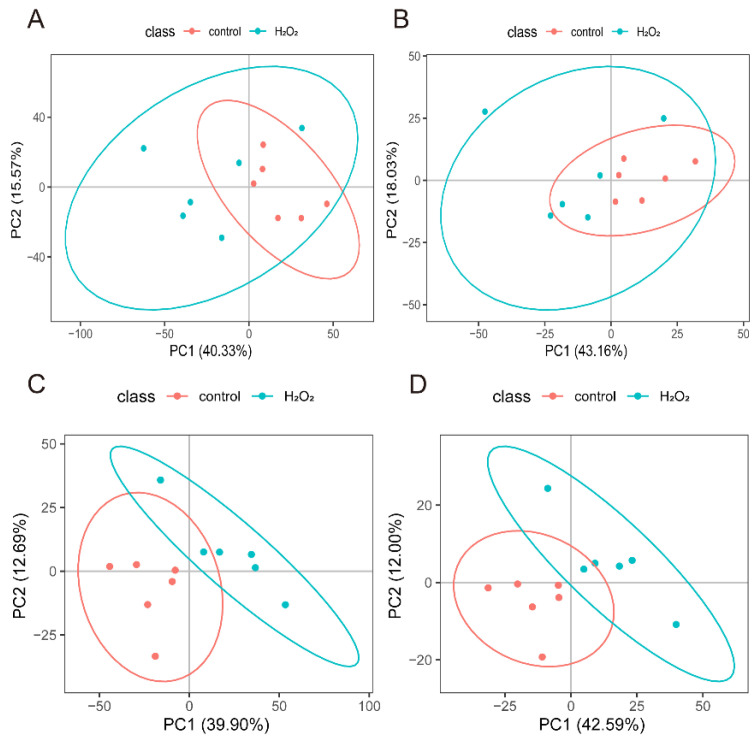
Screening of differential metabolites. (**A**) PCA analysis model from the POS mode. (**B**) PCA analysis model from the NEG mode. (**C**) PLS-DA analysis model from the POS mode. (**D**) PLS-DA analysis model from the NEG mode. POS, positive ion mode; NEG, negative ion mode; principal component analysis; PLS-DA, Partial least squares-discriminant analysis.

The POS- and NEG-specific metabolites (Figure 5A,B) were analysed separately using PCA, which showed a distinct separation between the H_2_O_2_ treatment and control groups. PLS-DA further confirmed that the optimal differentiation between the H_2_O_2_ treatment and the control groups was achieved in both the POS (Figure 5C) and NEG (Figure 5D).

### 3.8. Analysis of Differential Metabolites

**Figure 6:**
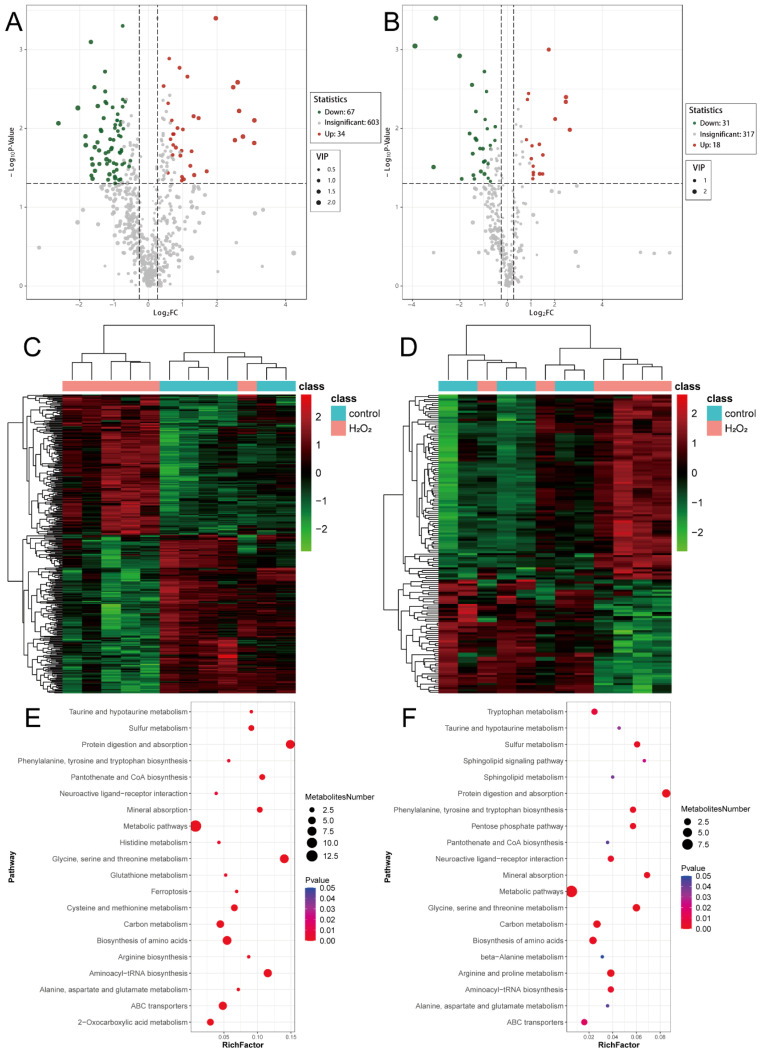
Analysis of differential metabolites. (**A**) Volcano map from the POS mode. (**B**) Volcano map from the NEG mode. (**C**) The heatmap from the POS mode. (**D**) The heatmap from the NEG mode. (**E**) Metabolic pathway analysis from the POS mode. (**F**) Metabolic pathway analysis from the NEG mode. POS, positive ion mode; NEG, negative ion mode.

Using the previously defined criteria for differential metabolite screening, 101 differential metabolites were identified in the POS, with 34 upregulated and 67 downregulated metabolites (Figure 6A). In the NEG, 49 metabolites were significantly differentially expressed, including 18 upregulated and 31 downregulated metabolites (Figure 6B). Hierarchical clustering of metabolites specific to the POS (Figure 6C) and NEG (Figure 6D) showed distinct expression patterns between the H_2_O_2_ treatment and control groups. Metabolic pathway enrichment analysis revealed that both the POS- (Figure 6E) and NEG-specific (Figure 6F) metabolites were enriched in pathways related to protein digestion and absorption; glycine, serine, and threonine metabolism; amino acid biosynthesis; and carbon metabolism. These findings suggest that H_2_O_2_ treatment significantly affects glycine, serine, and threonine metabolism. The detailed information for which is provided in Supplementary Files S11 and S12.

### 3.9. Correlation Cluster and PLS-DA Analyses

**Figure 7:**
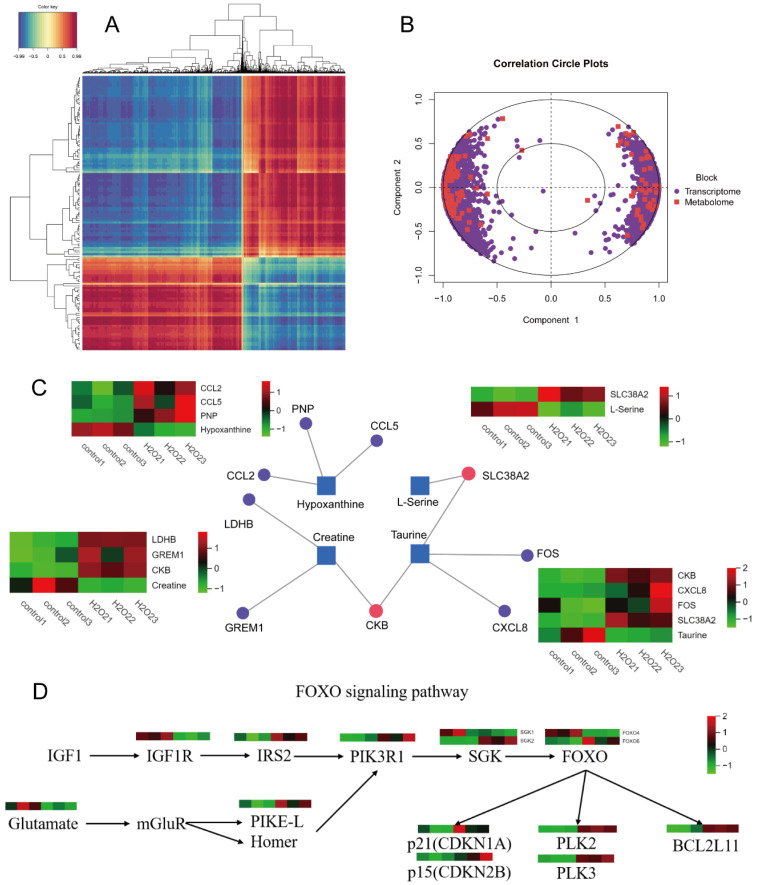
Correlation cluster analysis. (**A**) Heat map of correlation clustering. (**B**) Circle graph of correlation concentric. (**C**) Interactive network analysis diagram. (**D**) FOXO signalling pathway correlation analysis diagram.

The transcriptome and metabolome correlation clustering heatmap is presented in Figure 7A. In Figure 7B, the concentric circles represent the correlations between differential genes and metabolites. If the angle between a differential gene and metabolite was acute (<90°), the correlation was considered positive, whereas an obtuse angle (>90° but <180°) indicated a negative correlation. The length of the line from the centre of the circle to the differential genes and metabolites indicates the strength of the relationship; the longer lines represent a stronger relationship, and shorter lines indicate a weaker relationship.

### 3.10. Interaction Network and KEGG Pathway Association Analyses

Differential genes and metabolites were uploaded to MetaboAnalyst 5.0 for gene-metabolite interaction network analysis. The results showed that taurine interacted with *SLC38A2*, *FOS*, *CXCL8*, and *CKB*, exhibiting opposite expression trends. Creatine interacted with *CKB*, *LDHB*, and *GREM1*, exhibiting opposite expression trends. L-serine interacted with *SLC38A2*, exhibiting opposite expression trends. Hypoxanthine also interacted with *PNP*, *CCL2*, and *CCL5*, exhibiting opposite expression trends. The results are presented in Figure 7C, where the squares represent the differential metabolites, circles represent the differential metabolic genes, purple dots indicate the interactions between genes and a single metabolite, and the red dots represent interactions between genes and multiple metabolites. Functional annotation and pathway enrichment of DE mRNAs revealed significant enrichment in the FOXO signalling and mineral absorption pathways. The correlation analysis of the FOXO signalling pathway is shown in Figure 7D.

## 4. Discussion

In mammalian ovaries, the fate of follicles is determined by the state of the follicular GCs. The proliferation and differentiation of GCs drive follicular maturation and ovulation, whereas their apoptosis and degeneration lead to follicular atresia. Apoptosis of GCs is a critical event in follicle selection and development. It is influenced by various factors, and in particular by oxidative stress. In the present study, using H_2_O_2_-treated porcine GCs, a cellular oxidative stress model was established. Transcriptome sequencing and untargeted metabolomics were employed to identify the mRNAs and metabolites critical to oxidative stress in porcine GCs.

Porcine GCs provide an advantageous in vitro model for oxidative stress research. Porcine ovaries, readily available as slaughterhouse byproducts, are inexpensive and easily accessible, while sharing a high degree of similarity with human folliculogenesis. Consequently, porcine GCs are widely used as surrogate models for elucidating fundamental mechanisms of oxidative stress and for evaluating the protective effects of antioxidants. In comparison, bovine ovaries can also be collected from slaughterhouses, but their acquisition is more variable, being influenced by breed and physiological status. Bovine granulosa cells partially mimic human follicular dynamics and steroidogenic patterns, with research predominantly focusing on heat stress and reproduction-related impairments, which are of significant relevance in livestock production [18]. Human GCs, obtained from follicular fluid or oocyte retrieval during in vitro fertilization (IVF), represent the model most closely aligned with clinical pathological states. Despite the limited sample availability, human GCs provide the highest translational significance and are extensively employed in studies related to polycystic ovary syndrome (PCOS), infertility, and diminished ovarian reserve [19].

To construct the oxidative stress model, porcine follicular GCs were treated with exogenous H_2_O_2_. Considering the presence of catalase and other antioxidant enzymes in vivo, the concentration of H_2_O_2_ applied in vitro needs to be substantially higher than the physiological levels to induce oxidative stress. Under physiological conditions, intracellular H_2_O_2_ levels typically range from 1 to 10 nM; however, concentrations exceeding 100 nM have been reported to cause biomolecular damage and trigger oxidative stress [8]. In this experiment, the cytotoxic effects of H_2_O_2_ on cells exhibited a dose- and time-dependent relationship [4]. When porcine GCs were exposed to a low concentration (50–100 µM) of H_2_O_2_ for 2 h, the cells experienced initial oxidative stress stimulation, characterized by an elevation in intracellular ROS levels that did not reach a lethal threshold. During this phase, the cells activated antioxidant defense mechanisms—such as glutathione synthesis and upregulation of SOD—which partially mitigated oxidative damage, thereby maintaining relatively high cell viability. When the treatment duration was extended to 4 h, ROS accumulation reached its peak, and the antioxidant defense system was likely transiently exhausted. As a result, oxidative damage became more pronounced, apoptotic cell death increased, and overall cell viability reached its lowest point. After 6 h of exposure, due to the short half-life of exogenous H_2_O_2_ and the catalytic decomposition by intracellular enzymes such as CAT and glutathione peroxidase (GPx), the residual H_2_O_2_ was reduced, allowing partial cellular recovery. Meanwhile, the surviving cells exhibited enhanced activation of antioxidant response elements—such as the MAPK and Nrf2 signaling pathways—which promoted repair processes, leading to a subsequent increase in cell viability [20].

In general, low concentrations of H_2_O_2_ (<100 µM) typically induce only mild ROS elevation and reversible oxidative damage, whereas high concentrations (>500 µM) cause extensive cell death or complete loss of viability. In the present study, exposure of porcine granulosa cells to 300 µM H_2_O_2_ for 4 h resulted in approximately 50% cell survival. This threshold is widely recognized as a representative condition for oxidative stress induction, striking a balance between measurable cellular damage and sufficient viability for further analysis. When the survival rate is around 50%, it indicates that the system has undergone significant oxidative stress while retaining enough viable cells to support downstream assay, such as ROS quantification, signaling pathway analysis, and antioxidant enzyme expression. A survival rate higher than 50% suggests insufficient oxidative challenge and weak stress signaling, whereas a rate lower than 50% reflects irreversible damage leading to massive cell death. Therefore, a 50% survival rate is considered an optimal balance point for studying oxidative stress and cellular defense mechanisms [21]. ROS levels were measured following treatment, and a significant increase was observed in the H_2_O_2_-treated group compared with controls. Morphological assessment under microscopy revealed serrated cell edges, further confirming oxidative stress induction. KEGG pathway analysis revealed that the DE mRNAs were significantly enriched in multiple signaling pathways associated with cell proliferation and apoptosis, including the TNF and p53 signaling pathways. Tumor necrosis factor (TNF) is a key cytokine that, upon binding to its receptor TNFR1, can activate downstream caspase-8 and caspase-3, subsequently alter mitochondrial membrane permeability, and trigger the release of cytochrome c from mitochondria. Once released into the cytoplasm, cytochrome c activates caspase-9 and caspase-3, thereby initiating the apoptotic cascade. In addition, TNF can promote the generation of ROS within mitochondria. The accumulation of ROS not only disrupts cellular metabolism but also facilitates apoptosis. Excessive ROS can damage mitochondria, further promote cytochrome c release and the initiation of apoptosis. Moreover, ROS can enhance TNF signaling through a positive feedback mechanism, thereby amplifying apoptotic signaling [22]. p53 is a crucial intracellular tumor suppressor that induces apoptosis by upregulating pro-apoptotic proteins such as BAX and PUMA, which alter mitochondrial membrane permeability and promote the release of apoptogenic factors, including cytochrome c, from mitochondria into the cytoplasm, subsequently activating the caspase cascade and ultimately leading to apoptosis [23]. Additionally, p53 can facilitate apoptosis by downregulating anti-apoptotic proteins such as BCL-2 and BCL-xL. Beyond its role in DNA damage repair and cell cycle regulation, p53 also influences cellular energy metabolism. For example, p53 can modulate mitochondrial respiratory chain activity by regulating key metabolic genes (e.g., Cox4i1, PGC-1α), thereby controlling mitochondrial energy supply. Furthermore, p53 can regulate the activity of mitochondrial electron transport chain (ETC) complexes, thus affecting ATP synthesis and ROS production. As a byproduct of mitochondrial metabolism, ROS production is increased upon p53 activation, ultimately resulting in oxidative damage and apoptosis [24]. Thus, DE mRNAs play specific roles in the regulation of apoptosis in porcine GCs.

Glycine, serine, and threonine metabolisms are closely associated with oxidative stress. Glycine has been proven to have antioxidant properties because it scavenges free radicals and protects cells from oxidative damage [25]. Glycine is one of the end products of protein degradation, as well as a precursor for protein synthesis [26]. Additionally, glycine is metabolized by glycine cleavage enzyme to produce end products such as carbon dioxide, ammonia, and one-carbon units. These one-carbon units may be transferred to the tricarboxylic acid (TCA) cycle through interaction with tetrahydrofolate (THF), ultimately influencing the synthesis of oxaloacetate [27]. Oxaloacetate is a crucial intermediate in the TCA cycle, playing a significant role in cellular energy production and amino acid metabolism. It is not only essential for energy production but also plays an important role in gluconeogenesis and amino acid synthesis [28]. Serine serves as a major intracellular source of one-carbon units, which are released through its conversion to glycine by mitochondrial serine hydroxymethyltransferase (SHMT). These one-carbon units are essential for critical cellular processes such as DNA synthesis and methylation regulation [29]. Serine deficiency disrupts mitochondrial morphology, membrane potential, and the dynamics of fission and fusion, thereby compromising mitochondrial functional stability. Additionally, serine supports mitochondrial membrane integrity and energy production by contributing to lipid biosynthesis, replenishing tricarboxylic acid (TCA) cycle intermediates, and generating NADPH [30]. The NADPH produced via serine metabolism plays a pivotal role in the detoxification of mitochondrial reactive oxygen species (ROS), thereby mitigating oxidative stress within the cell [31]. Serine and threonine act as precursors in the synthesis of glutathione (GSH) [32], GSH is one of the most critical antioxidants within mitochondria and plays an essential role in protecting cells against oxidative stress [33]. GSH can be oxidised by ROS to form oxidised glutathione, and excess levels of ROS in cells are eliminated through the ascorbate-GSH cycle [34]. Glycine, serine, and threonine metabolism promote antioxidant defense mechanisms and indirectly influence ROS production. These metabolic pathways participate in mitochondrial function and energy metabolism [35]. Mitochondria are the primary sources of ROS, and disruptions of these pathways can compromise mitochondrial function, resulting in elevated ROS production and oxidative stress [36]. As a result, the metabolism of glycine, serine, and threonine is closely linked to the regulation of the cellular redox balance and oxidative stress. Dysregulation of these metabolic pathways may lead to oxidative stress, while their proper regulation may alleviate oxidative damage and aid the maintenance of cellular homeostasis.

In the analysis of gene-metabolite interaction networks, taurine, creatine, L-serine, and hypoxanthine were found to be pivotal metabolites that are involved in numerous metabolic pathways and play crucial roles in cellular function and oxidative stress responses. Taurine, an antioxidant synthesised from cysteine and methionine via the hepatic transsulphuration pathway, protects cells from oxidative stress by scavenging ROS [37]. It also boosts the activity of antioxidant enzymes, including superoxide dismutase and catalase [38], and prevents calcium overload-induced cellular damage by regulating the intracellular calcium levels [39]. Creatine, primarily synthesised from arginine, glycine, and methionine in the liver, kidneys, and pancreas, is essential for cellular energy metabolism [40]. As part of the phosphagen system, one of the body’s three primary energy supply systems, creatine phosphate provides a readily available source of ATP through high-energy phosphate molecules, thereby protecting cells from oxidative stress-induced energy depletion [41]. In addition, creatine mitigates oxidative damage to cellular components by scavenging free radicals and lipid peroxides [42]. L-serine, a precursor for GSH synthesis, is generated via the glycolytic pathway from 3-phosphoglycerate [43]. GSH, a primary intracellular antioxidant, plays a core role in cellular antioxidant defense by neutralising ROS and protecting cells from oxidative stress [44,45]. L-serine also protects against oxidative stress-induced neuronal injury by enhancing antioxidant defense mechanisms and reducing oxidative stress markers [46]. Hypoxanthine is converted to xanthine through xanthine oxidase, with uric acid being the final product of purine metabolism, a process that also generates ROS. Elevated levels of hypoxanthine result in increased ROS production, which, in turn, triggers endothelial dysfunction and apoptosis by modulating the expression of apoptosis-related proteins [47,48]. These metabolites are essential for cellular metabolism and energy homeostasis.

The KEGG pathway enrichment analysis revealed a significant association between the DE genes and metabolites with the FOXO signalling, and mineral absorption pathways. The Forkhead box O (FOXO) signaling pathway serves as a central regulatory system in cellular stress responses, metabolic regulation, and longevity control. Glutamate, an essential metabolite in cellular metabolism, is involved in the citric acid cycle and acts as a precursor to produce various amino acids and neurotransmitters. It enters cells through metabotropic glutamate receptors (mGluRs), and research has shown that phosphoinositide 3-kinase enhancer-L (PIKE-L) binds to Homer in a physiological context. Activation of Group I mGluRs promotes the formation of the mGluR-Homer-PIKE-L complex, which, in turn, activates the phosphatidylinositol-3-kinase (PI3K) signalling pathway [49]. PI3K, a lipid kinase, produces phosphatidylinositol-3,4,5-trisphosphate, a secondary messenger that recruits AKT to the plasma membrane. AKT is subsequently phosphorylated and activated by phosphoinositide-dependent kinases (PDK1 and PDK2) on the cell membrane. Activated AKT then phosphorylates various substrates, playing essential roles in cell proliferation, survival, growth, and angiogenesis [50].

FOXO transcription factors are negatively regulated by the PI3K/AKT signalling pathway, which inhibits cell proliferation. For instance, the PI3K pathway regulates keratinocyte proliferation by downregulating FOXO through activation of targets such as AKT [51]. During apoptosis, FOXO transcription factors activate both mitochondrion-dependent and -independent pathways, driving cell death by inducing the expression of death receptor ligands, such as Fas and TNF-related apoptosis-inducing ligands, as well as pro-apoptotic proteins, such as BCL-XL, bNIP3, and Bim from the BCL-2 family, thereby promoting apoptotic processes [52]. Additionally, FOXO proteins play a key role in oxidative stress-associated apoptosis, a process initiated by the release of ROS into the cellular environment [53]. The FOXO family transcription factor FOXO3 regulates the expression of downstream targets such as PGC-1α (Peroxisome proliferator-activated receptor gamma coactivator 1-alpha), thereby promoting mitochondrial biogenesis and functional recovery, and influencing mitochondrial synthesis [54]. In parallel, FOXO3 facilitates mitophagy by activating the PINK1/Parkin pathway, enabling the clearance of damaged mitochondria and maintaining mitochondrial quality and function [55]. Moreover, FOXO cooperates with SIRT1 to regulate the NAD^+^/SIRT1/FOXO axis, thereby controlling energy metabolism and mitochondrial performance [56]. Additionally, FOXO3a activation enhances the expression of antioxidant enzymes such as SOD2 and CAT, which mitigates mitochondrial-derived ROS and alleviates oxidative stress [57].

Galactose and glucose enter cells through the cell membrane and are metabolized via carbohydrate and protein metabolic pathways as part of the mineral absorption process. Within this pathway, the key gene heme oxygenase 1 (HMOX1) plays a central role in iron metabolism. *HMOX1* catalyzes the degradation of heme to generate Fe^2+^. Excessive iron accumulation enhances the Fenton reaction, leading to the generation of ROS and inducing lipid peroxidation, which consequently triggers ferroptosis [58]. Moreover, overexpression of *HMOX1* in diabetes and sickle cell disease exacerbates iron overload, resulting in ferroptosis of endothelial or cardiomyocytes [59]. Subsequently, ferroportin 1 (FPN1) in this pathway is responsible for exporting Fe^2+^ out of the cell. Its expression is regulated by the β-catenin/TCF4–SLC7A11/FPN1 axis, which reduces ROS accumulation and alleviates ferroptosis [60]. Metallothioneins (MTs), as crucial components of the mineral absorption pathway, play vital roles in maintaining Cu^2+^ and Zn^2+^ homeostasis. Disruption of copper and zinc metabolism can impair the cellular redox balance, promote ROS accumulation, and potentially aggravate ferroptosis [61]. The zinc transporter 1 (ZnT1, SLC30A1), located on the plasma membrane, exports Zn^2+^ from the cell, thereby regulating intracellular zinc concentrations [62]. Studies have shown that Zn^2+^ overload can activate ROS production via the MAPK signaling pathway (ERK1/2, JNK1/2/3, and p38 MAPK), inducing necrosis and apoptosis, and exhibiting crosstalk with the oxidative stress mechanisms of ferroptosis [63]. These findings suggest that key molecules in the mineral absorption pathway (HMOX1, FPN1, ZnT1, and MTs) are closely associated with the occurrence and regulation of ferroptosis through their effects on the metabolism of iron, copper, and zinc ions. In multi-omics integrative analyses, the relationships between key metabolites such as taurine, creatine, L-serine, and hypoxanthine and DEmRNAs are not simple linear causal associations but are instead mediated through complex transcriptional regulatory networks. Specifically, variations in metabolite levels often arise from the transcriptional regulation of metabolic enzyme–encoding genes by transcription factors (TFs), thereby influencing metabolic flux and homeostasis. Previous studies have demonstrated that several transcription factors, including Nrf2, ATF4, PGC-1α, and p53, function as master regulators in this process. Under oxidative stress, Nrf2 upregulates genes such as *CDO1* and *CSAD*, promoting taurine biosynthesis and enhancing antioxidant capacity. PGC-1α regulates *GATM* and *GAMT* to facilitate creatine metabolism and maintain energy homeostasis. ATF4, in response to amino acid stress, induces the expression of *PHGDH* and *PSAT1* to strengthen serine and one-carbon metabolism. Meanwhile, p53 modulates *HPRT1* and *XDH* to sustain purine metabolic balance and participate in DNA repair processes. These transcription factors not only control the expression of metabolic enzyme genes but also sense cellular metabolic states through feedback mechanisms, achieving bidirectional coordination between gene expression and metabolic flux. Collectively, these TFs can be regarded as central hubs linking the transcriptome and metabolome, elucidating how gene expression changes systematically drive dynamic remodeling of metabolite levels.

In conclusion, the integrated transcriptomic and metabolomic analyses presented in this study provide comprehensive insights into the regulatory landscape of oxidative stress in porcine granulosa cells. The findings highlight that oxidative damage induced by H_2_O_2_ triggers a coordinated transcriptional and metabolic response involving redox homeostasis, mitochondrial function, and cell fate determination. Key signaling pathways such as TNF, p53, and FOXO, together with metabolic adaptations in amino acid, energy, and mineral metabolism, collectively shape the cellular defense against oxidative injury. These results not only deepen our understanding of the molecular mechanisms underlying follicular atresia but also offer valuable references for developing targeted interventions to maintain granulosa cell function and improve ovarian follicular health.

## 5. Conclusions

This study established an oxidative stress model in porcine granulosa cells (GCs) and explored its molecular mechanisms through integrated transcriptomic and metabolomic analyses. A total of 328 differentially expressed genes were enriched in apoptosis-related pathways (e.g., TNF and p53), while 150 metabolites were mainly involved in glycine, serine, threonine, and carbon metabolism. Multi-omics integration identified taurine, creatine, L-serine, and hypoxanthine as key metabolites, with both genes and metabolites enriched in the FOXO signaling and mineral absorption pathways. Overall, oxidative stress disrupts redox balance in GCs by inducing transcriptional and metabolic reprogramming, thereby affecting apoptosis, antioxidant defense, and energy metabolism.

## Data Availability

The data used to support the findings of this study are available from the corresponding author upon reasonable request. The datasets generated and/or analysed during the current study are available in the NCBI BioProject at https://dataview.ncbi.nlm.nih.gov/object/PRJNA1244716 (accessed on 19 October 2025), with the accession number PRJNA1244716.

## Supplementary Materials

The following supporting information can be downloaded at: https://www.mdpi.com/article/10.3390/biology14111519/s1, Files S1: Primer sequences; Files S2: Total RNA integrity of porcine GCs control group and H_2_O_2_ treatment group; Files S3: Sequence comparison of sample sequencing data with the selected reference genome; Files S4: Information of DE mRNAs; Files S5: Information of mRNAs; Files S6: Cluster analysis of the DE mRNAs; Files S7: KEGG pathways of DE mRNAs; Files S8: Data quality control of QC samples; Files S9: Information of POS metabolites; Files S10: Information of NEG metabolites; Files S11: KEGG pathways of POS metabolites; Files S12: KEGG pathways of NEG metabolites.

## Abbreviations

AKT/PKB	protein kinase B
ATF4	Activating Transcription Factor 4
BGI	Borland Graphics Interface
BTG1	B-Cell Translocation Gene 1, Anti-Proliferative
BPC	Base peak chromatogram
CDO1	Cysteine Dioxygenase Type 1
CSAD	Cysteine Sulfinic Acid Decarboxylase
CV	Coefficient of variation
DE	Differentially expressed
DEGseq	Identify Differentially Expressed Genes from RNA-seq data
ERK	Extracellular regulated protein kinases
ETC	electron transport chain
FC	Fold-change
FOXO	Forkhead box O
FPKM	Fragments Per Kilobase of exon model per Million mapped fragments
FPN1	ferroportin 1
GATM	Glycine Amidinotransferase
GAMT	Guanidinoacetate N-Methyltransferase
GCs	Granulosa cells
GO	Gene Ontology
GSH	Glutathione
H_2_O_2_	Hydrogen peroxide
HISAT	Hierarchical Indexing for Spliced Alignment of Transcripts
HMDB	Human Metabolome Database
HMOX1	Haeme Oxygenase 1
HPRT1	Hypoxanthine Phosphoribosyltransferase 1
JNK	Jun N-terminal Kinase
KEGG	Kyoto Encyclopedia of Genes and Genomes
LIPG	Lipase G, Endothelial Type
MAPK	Mitogen activated protein kinase
mGluRs	metabotropic glutamate receptors
MTs	Metallothioneins
NEG	Negative ion mode
NF-κB	Nuclear factor kappa-B
Nrf2	Nuclear Factor Erythroid 2–Related Factor 2
p53	Tumor Protein p53
PCA	Principal Coordinates Analysis
PDK	Phosphoinositide-dependent kinases
PGC-1α	Peroxisome proliferator-activated receptor gamma coactivator 1-alpha
PHGDH	Phosphoglycerate Dehydrogenase
PI3K	Phosphatidylinositol-3-kinase
PLK2	Polo Like Kinase 2
PIKE-L	Phosphoinositide 3-kinase enhancer-L
PLS-DA	Partial least squares-discriminant analysis
POS	Positive ion mode
PSAT1	Phosphoserine Aminotransferase 1
PSPH	Phosphoserine Phosphatase
QC	Quality control
rCCA	regularised Canonical Correlation Analysis
ROS	Reactive oxygen species
SIRT1	Sirtuin 1
SLC40A1	Solute Carrier Family 40 Member 1 (also known as Ferroportin 1)
TCA	Tricarboxylic acid
THF	tetrahydrofolate
TNF	Tumour necrosis factor
TP53INP1	Tumor Protein p53 Inducible Nuclear Protein 1
TXNIP	Thioredoxin Interacting Protein
XDH	Xanthine Dehydrogenase
ZnT1	Zinc transporter 1
