# Supplementary material for: Combined Transcriptome and Metabolome Analyses of Oxidative Stress Regulatory Mechanism in Porcine Follicular Granulosa Cells
Source: Biology (Basel). 2025 Oct 30;14(11):1519. doi: 10.3390/biology14111519 (PMC12650230; doi:10.3390/biology14111519)
Supplement: Supplementary file 1 [file biology-14-01519-s001.zip › supplementary file.pdf]

Additional materials

- File name: Additional file S1
- File format:
- Title of data: Primer sequences
- Description of data: Primer sequence of PLK2, LIPG, ATF4, TP53INP1, HMOX1, BTG1, SLC40A1, TXNIP and GAPDH

PLK2: Forward primer: ATCCTTGTTGGCTCCTGCTA

Reverse primer: GTGGAAATCGGGAACTGTATG

LIPG: Forward primer: CACCCTTTACGGCACCAAT

Reverse primer: ACAGGCTGAACCAGGACTGA

ATF4: Forward primer: TTCAAACCTCATGGGTTCTCC

Reverse primer: CCATTTTCTCCACCATCCAGT

TP53INP1: Forward primer: GAGCACCCTTCCGTCTTTTC

Reverse primer: AGGACTGGTTTCCACCTTGAT

HMOX1: Forward primer: CACTCACAGCCCAACAGCAT

Reverse primer: TGGTACAAGGACGCCATCAC

BTG1: Forward primer: GTTCAGGCTTCTCCCAAGTG

Reverse primer: GGTGCTGTTTTGGGTGCTAC

SLC40A1: Forward primer: ACCCCTGCTCTAGCTGTGAA

Reverse primer: TGGATGTCAGGGTCTTTCTCA

TXNIP: Forward primer: CGGTCAGAGGCAATCACATT

Reverse primer: TCTTGGAGCCAGGGACACTA

GAPDH: Forward primer: GATCCCGCCAACATCAAAT

Reverse primer: TCACGCCCATCACAAACAT

- File name: Additional file S2
- File format:
- Title of data: Total RNA integrity of porcine GCs control group and H_2O_2 treatment group
- Description of data: (A, B, C) Total RNA integrity in control group. (D, E, F) Total RNA integrity in H_2O_2 treatment group.

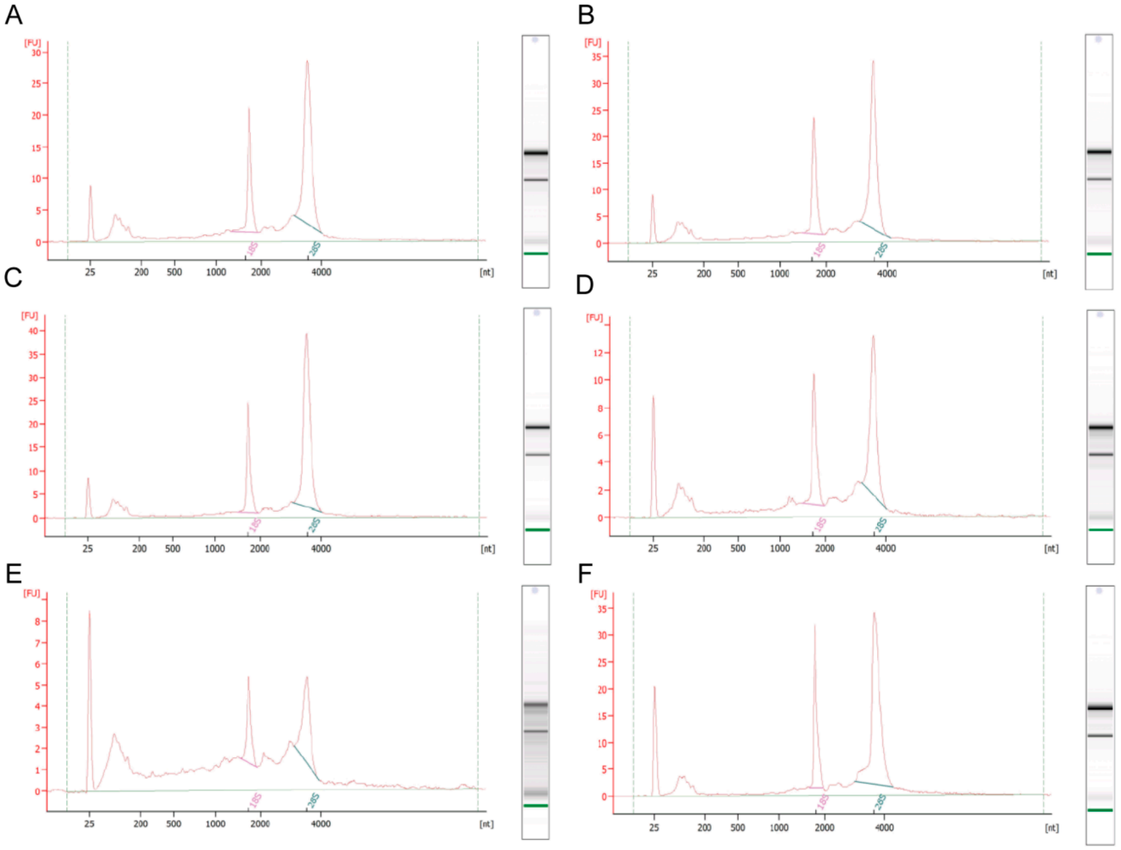

- File name: Additional file S3
- File format: xlsx
- Title of data: Sequence comparison of sample sequencing data with the selected reference genome
- Description of data: The Q20 and Q30 of three H_2O_2 treatment and control groups

- File name: Additional file S4
- File format: xlsx
- Title of data: Information of DE mRNAs
- Description of data: Gene ID, Gene Symbol, FPKM of samples, FC, p value, q value of DE mRNAs

- File name: Additional file S5
- File format: xlsx
- Title of data: Information of mRNAs
- Description of data: Gene ID, Gene Symbol, FPKM of samples, log2(FC), p value, q value of mRNAs

- File name: Additional file S6
- File format:
- Title of data: Cluster analysis of the DE mRNAs

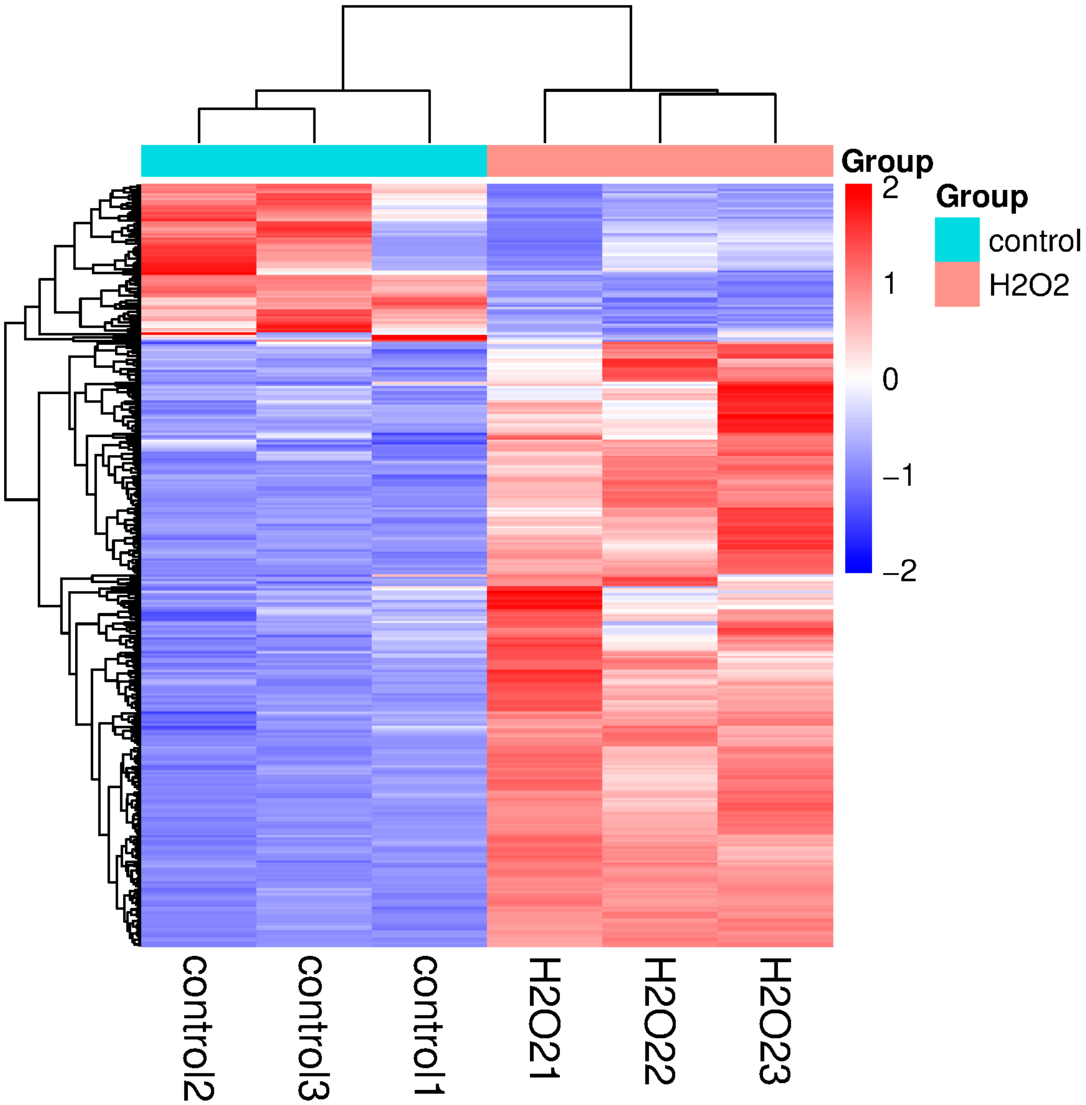

- File name: Additional file S7
- File format: xlsx
- Title of data: KEGG pathways of DE mRNAs
- Description of data: Pathway ID, Description, Gene ID, p value, p adjust, q value and count

- File name: Additional file S8
- File format:
- Title of data: Data quality control of QC samples.
- Description of data: (A) BPC overlay of QC samples from the POS mode. (B) BPC overlay of QC samples from the NEG mode. (C) PCA for all QC samples from the POS mode. (D) PCA for all QC samples from the NEG mode. (E) CV distribution in each QC sample from the POS mode. (F) CV distribution in each QC sample from the NEG mode.

BPC, base peak chromatogram; POS, positive ion mode; NEG, negative ion mode; QC, quality control; CV, coefficient of distribution; PCA, principal component analysis

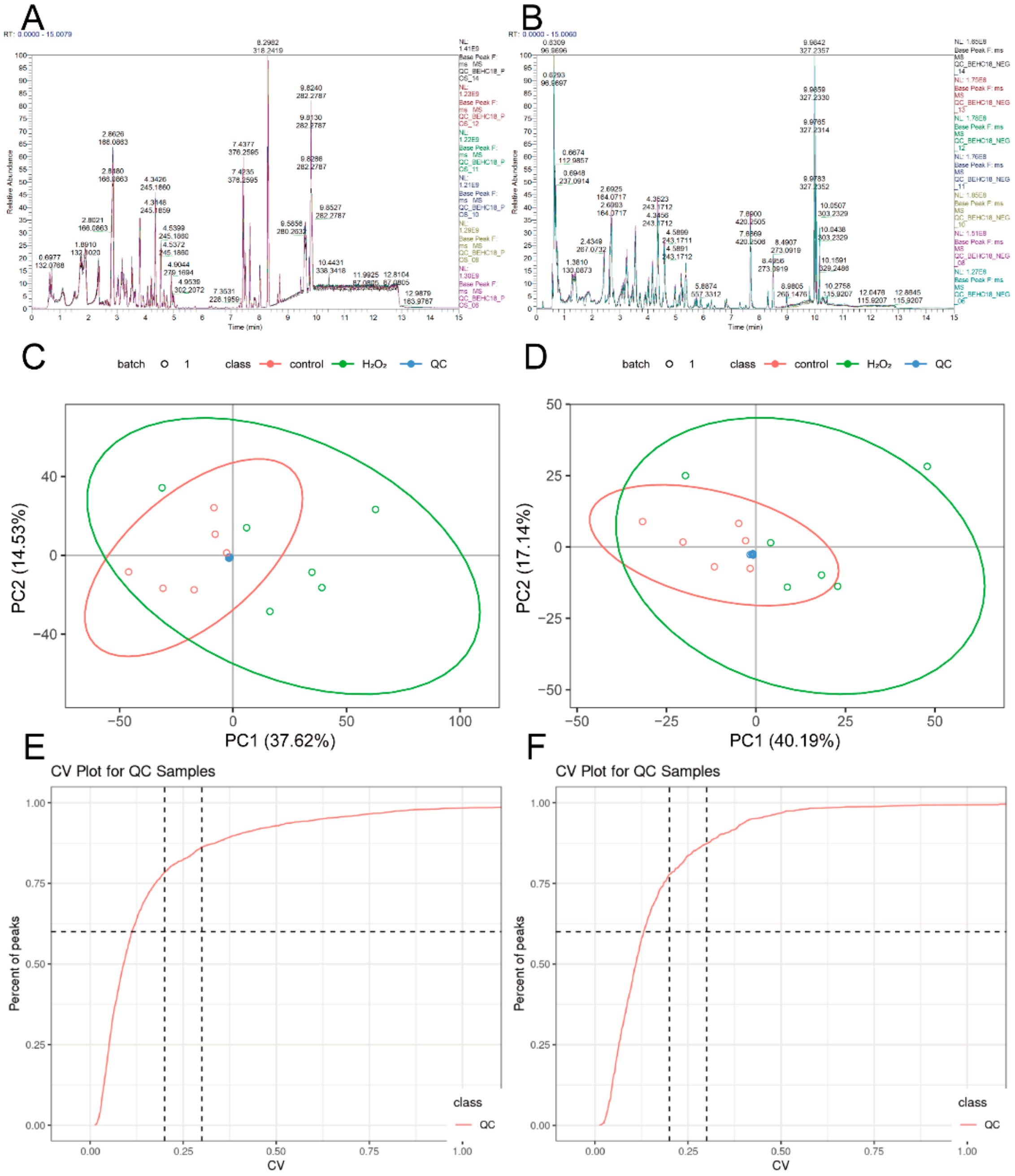

- File name: Additional file S9
- File format: xlsx
- Title of data: Information of POS metabolites

- Description of data: Compound ID, ratio(H_2O_2:control), p value, Name, Formula, Molecular Weight of metabolites

- File name: Additional file S10
- File format: xlsx
- Title of data: Information of NEG metabolites
- Description of data: Compound ID, ratio(H_2O_2:control), p value, Name, Formula, Molecular Weight of metabolites

- File name: Additional file S11
- File format: xlsx
- Title of data: KEGG pathways of POS metabolites
- Description of data: Pathway Name, Pathway ID, p value, KEGG Names and KEGG IDs

- File name: Additional file S12
- File format: xlsx
- Title of data: KEGG pathways of NEG metabolites
- Description of data: Pathway Name, Pathway ID, p value, KEGG Names and KEGG IDs
